# Identification of a Novel Signature and Construction of a Nomogram Predicting Overall Survival in Clear Cell Renal Cell Carcinoma

**DOI:** 10.3389/fgene.2020.01017

**Published:** 2020-09-04

**Authors:** Xiangkun Wu, Zhijian Zhao, Aisha Khan, Chao Cai, Daojun Lv, Di Gu, Yongda Liu

**Affiliations:** ^1^Department of Urology, Minimally Invasive Surgery Center, The First Affiliated Hospital of Guangzhou Medical University, Guangzhou, China; ^2^Guangdong Key Laboratory of Urology, Guangzhou Institute of Urology, Guangzhou, China; ^3^Department of Family Medicine, Yunshan Medical Hospital Shenzhen, Shenzhen, China

**Keywords:** clear cell renal cell carcinoma, Robust Rank Aggregation method, nomogram, TCGA, GEO, ICGC

## Abstract

**Background:**

Clear cell renal cell carcinoma (ccRCC) is the most common subtype of renal cell carcinoma (RCC), which accounts for majority of RCC-related deaths. It is clearly essential to further identify more novel prognostic signatures and therapeutic targets.

**Material and Methods:**

We identified differentially expressed genes (DEGs) between ccRCC and adjacent normal tissues in GEO database using a Robust Rank Aggregation (RRA) method. An mRNA signature (mRNASig) based on DEGs was developed using Cox and LASSO analysis in the TCGA database and validated in the ICGC database. Afterward, the influence of mRNASig mRNAs on the immune microenvironment in ccRCC was explored using comprehensive bioinformatics analysis.

**Results:**

A total of 957 robust DEGs were identified using the RRA method. mRNASig comprised CEP55, IFI44, NCF4, and TCIRG1 and was developed and validated to identify high-risk patients who had poorer prognosis than low-risk patients. A nomogram was also constructed based on mRNASig, AJCC stage, and tumor grade. The mRNASig were closely related to a variety of tumor-infiltrating lymphocytes, especially including CD8+ T cells, activated CD4+ memory T cells, regulatory T cells, activated NK cells, and resting NK cells. The mRNASig were also correlated positively with the expression of CTLA4, LAG3, PDCD1, TIGIT, and HAVCR2.

**Conclusion:**

We developed and validated mRNASig to assist clinicians in making personalized treatment decisions. Furthermore, CEP55, IFI44, NCF4, and TCIRG1 may be novel potential targets for future treatment of ccRCC.

## Introduction

The renal cell cancer (RCC) is a common malignant tumor of the urinary system with increasing incidence and accounts for 3% of all the new cancer cases (Siegel et al., 2020). According to the global cancer statistics of 2018, there were approximately 403,262 (2.2%) newly diagnosed cases and around 175,098 (1.8%) deaths due to RCC (Bray et al., 2018). The clear cell renal cell carcinoma (ccRCC) is the most common subtype of RCC and accounts for majority of RCC-related deaths (Hsieh et al., 2017). The ccRCC has an insidious onset and does not often show any early clinical symptoms. More than 30% of patients usually are at metastasis by the time they are diagnosed (Cairns, 2010). There are about 30% of the localized ccRCC patients with curative intent who eventually have recurrence or develop metastatic disease following radical or partial nephrectomy (Meskawi et al., 2012; Wolff et al., 2016; Hsieh et al., 2017). Therefore, it is essential to identify more novel prognostic signatures and therapeutic targets.

The open access of microarray and high-throughput sequencing data have allowed increasing number of researchers to use comprehensive bioinformatics such as mRNA (He et al., 2020), miRNA (Xiao et al., 2020), and lncRNA (Wu et al., 2019) to explore the novel signatures related to tumor progression and prognosis of ccRCC patients. However, the small sample size of individual studies and differences in sequencing platforms and lab protocols can render great variability among the studies. The Robust Rank Aggregation (RRA) method can solve this problem, which directly integrates the lists of differentially expressed genes (DEGs) analyzed by different datasets (Kolde et al., 2012) and identifies more robust cancer-related gene sets (Griffith et al., 2006). Besides, the combination of novel signatures with clinicopathological information may improve the prediction of prognosis in ccRCC patients, but this has not been widely applied in clinical practice (Chen et al., 2019a; Zhang et al., 2020). Thus, it is necessary to find more novel signatures through comprehensive bioinformatics to establish a more accurate nomogram than just the clinicopathological information.

In this study, we aim to

(a) Identify the robust DEGs between ccRCC and adjacent normal tissue using seven GEO datasets;
(b) Use the univariate Cox regression analysis and Least Absolute Shrinkage and Selection Operator method (LASSO) to develop the mRNA signature (mRNASig) that can predict the prognosis of ccRCC patients in the TCGA cohort and validate the mRNASig by internal validation in the TCGA cohort as well as external validation in ICGC cohort;
(c) Construct a nomogram by combining the signature and clinicopathological information;
(d) Explore the potential molecular mechanism and tumor immune microenvironment relevance of mRNASig mRNAs.

## Materials and Methods

### Data Acquisition and Processing

The matrix file series of microarray datasets were downloaded from the GEO dataset^1^. The selection criteria of microarray datasets were as follows: (1) human kidney tissue samples; (2) containing at least 10 ccRCC and adjacent normal tissue samples. However, the “Xenograft” and “cell line” were excluded from the study. Eventually, seven microarray datasets that met the above criteria were included in the study for DEG analysis: GSE16449, GSE17895, GSE36895, GSE40435, GSE53757, GSE66270, and GSE71963. We used the annotation files provided by the platform to match the probes with the gene symbols. If multiple probes matched a single gene, then the average of the multiple probes was considered to be the expression value of the gene.

In addition, the raw counts of ccRCC RNA-sequencing and corresponding clinical data were downloaded from the TCGA dataset^2^ and were analyzed in the study. The selection criteria of TCGA samples were as follows: (1) pathological diagnosis was ccRCC, (2) having complete clinical information, and (3) follow-up of more than 30 days. Finally, there were 517 ccRCC patients who were selected from the TCGA dataset and were randomly assigned to the cohort training and internal validation cohort to a ratio of 1:1. The raw counts of RNA-sequencing data were transformed into transcripts per million (TPM) values and were further log2-transformed (log2TPM) for subsequent analyses (Wagner et al., 2012). The mRNA matrix data expression profile and follow-up data were downloaded from the ICGC dataset as an external validation cohort^3^. There were a total of 91 ccRCC patients who were included in the external validation analysis.

### Identification of Robust DEGs

The DEGs between ccRCC and adjacent normal tissue were identified using “limma” package in R software (version 3.6.2). The RRA method based on “RobustRankAggregation” package was performed to integrate the DEGs of those seven microarray datasets to find the robust DEGs. In the RRA analysis, the genes with |log2FC| > 1 and false discovery rate (FDR) < 0.05 were considered robust DEGs.

### Function Enrichment Analysis of Robust DEGs

The GO enrichment analysis included biological processes (BPs), cellular components (CCs), molecular functions (MFs), and KEGG enrichment analysis of robust DEGs that were performed using “clusterprofiler” package. The value of FDR < 0.05 was considered statistically significant (Yu et al., 2012).

### Survival Analysis of Robust DEGs and Development of mRNASig

We constructed the mRNASig to predict the survival probability of ccRCC patients in the training cohort. Firstly, we performed univariate Cox regression analysis to identify the survival-related DEGs. The robust DEGs with HR (hazard ratio) ≠ 1 and *P* < 0.05 were considered as survival-related DEGs and were included in subsequent analyses. Secondly, the LASSO analysis was performed to further screen candidates’ DEGs with best predictive performance based on “glmnet” package and the 10-fold cross-validation was used to identify the optimal lambda value such that the error is limited within a minimum of 1 standard error (Friedman et al., 2010). Finally, the mRNASig was constructed based on LASSO coefficients (*L*_*i*_) derived from LASSO model multiplied with the relative expression levels of mRNAs (Exp*_*i*_*), $\text{Risk}\,\text{score}=\sum_{i=1}^{n}L_{i}\times {\text{Exp}}_{i}$.

The patients in the training cohort were divided into high- and low-risk groups based on the cutoff value of median risk score. The Kaplan–Meier analysis with log-rank test was performed to compare the survival differences between high- and low-risk groups. The area under the curve (AUC), time-dependent receiver operating characteristic (tROC) curve, and concordance index (C-index) were used to evaluate the prognostic performance of mRNASig. The prognostic performance of clinical variables such as age, AJCC-stage, T-stage, N-stage, M-stage, and grade were used as controls.

### Internal and External Validation of mRNASig

To evaluate the potential and applicability of mRNASig, the validation was done in the internal validation cohort, entire cohort, and external validation cohort. In the internal validation cohort, the risk score of each patient was calculated using the same formula and the same cutoff value. The patients were then divided into high- and low-risk groups and the Kaplan–Meier analysis with log-rank test was performed to compare the survival differences between the groups.

### Identification of Independent Prognostic Variables of ccRCC Patients

To identify the independent prognostic value of mRNASig and other clinical variables, the univariate and multivariate Cox regression analyses were performed in the training cohort, validation cohort, and entire cohort on mRNASig and clinical variables (including age, AJCC-stage, T-stage, N-stage, M-stage, and grade). In the multivariate Cox regression analysis, the stepwise method was used to screen variables. To investigate the predictive value of the signature in different subgroups stratified by clinical variables, we used Kaplan–Meier analysis to access overall survival difference between high- and low-risk groups of different subgroups in the entire cohort.

### Construction and Validation of a Nomogram

A nomogram was constructed based on mRNASig and clinical variables using a stepwise Cox regression model to predict the probability of OS in ccRCC at 1, 3, and 5 years in the entire cohort. Furthermore, a clinical model was constructed based on clinical variables through a stepwise Cox regression model to evaluate whether mRNASig could improve the predictive performance of the clinical variables. Additionally, the net benefits derived from the use of clinical model, mRNASig, and nomogram were determined by using the decision curve analysis (Vickers and Elkin, 2006).

### Comprehensive Bioinformatics Analysis of mRNASig mRNAs

The differences in mRNA expression level and protein expression of mRNAs between ccRCC and adjacent normal tissue were validated in the TCGA dataset using the UALCAN website^4^. The data of protein expression analysis provided by UALCAN was from the Clinical Proteomic Tumor Analysis Consortium (CPTAC) Confirmatory/Discovery dataset (Chen et al., 2019b). Moreover, we also used the TISIDB website to analyze the association between the mRNA expression level and clinical traits, including the overall survival analysis, cancer staging, and tumor grading^5^.

The sensitivity (true positive rate) and specificity (true negative rate) of mRNASig mRNAs for ccRCC diagnosis were evaluated by ROC curve analysis, and the AUC was calculated using “pROC” package in R software. The data of ccRCC and normal tissues protein expression was downloaded from the CPTAC database^6^ and was used to analyze the efficacy of the corresponding proteins in diagnosing ccRCC.

The Gene Set Enrichment Analysis (GSEA) was conducted to explore the potential molecular mechanisms of mRNASig mRNAs. The TCGA ccRCC dataset was divided into low- and high-expression groups according to the median expression of mRNAs. The GSEA was performed in GSEA 4.0.0 software based on the Molecular Signatures Database V7.0 (MSigDB) and was used to explore the enriched KEGG pathways according to the reference gene set “c2.cp.kegg.v6.2.symbols.gmt.” |NES| > 1 and FDR < 0.05 were regarded as statistically significant.

### Tumor Immunity Analysis of the Signature mRNAs

The TIMER web server^7^ was used to evaluate the relationship between mRNASig mRNAs expression levels and six tumor-infiltrating lymphocytes (TILs: B cells, CD4+ T cells, CD8+ T cells, neutrophils, macrophages, and dendritic cells). The abundance of the six TILs was estimated by TIMER algorithm (Li et al., 2016; Li et al., 2017). The co-relations were further investigated using the CIBERSORT website^8^, which is a deconvolution algorithm to estimate TILs of complex tissues based on gene expression and was used to measure the abundance of 22 TILs (Chen et al., 2018). The input data included TPM data of TCGA ccRCC and gene signature matrix (LM22), which were used to distinguish the 22 types of TIL. The permutation parameter was set as 1000 to create a meaningful *P-*value. The output included a fraction of estimated TIL types in each sample and a *P-*value for the global deconvolution of each sample. Therefore, to ensure a high reliability of the estimation, a *P-*value < 0.05 was set up.

In addition, immune, stromal, and estimate scores of each TCGA ccRCC patient were obtained from the ESTIMATE website^9^ to evaluate the association of mRNASig mRNAs and the tumor microenvironment. The ESTIMATE algorithm is based on single sample GSEA to evaluate tumor purity (Yoshihara et al., 2013). The expression levels of immune checkpoints have become a biomarker for selecting the ccRCC patients for immunotherapy. Therefore, we assessed the correlation between mRNASig mRNAs and expression levels of critical immune checkpoints (CTLA4, LAG3, PDCD1, TIGIT, and HAVCR2) using the TISIDB website.

### Patients Samples

Primary ccRCC specimens and adjacent normal renal tissues were obtained from 36 patients diagnosed with ccRCC who underwent surgical resection at the Urology Department of the First Affiliated Hospital of Guangzhou Medical University between January 2018 and November 2019. ccRCC tissues were stocked at −80°C for further processing. All the patients did not receive preoperative treatment, such as chemotherapy or radiation. All the patients arranged their written informed consent to participate in this study, and the study was approved by the Ethics Committee of First Affiliated Hospital of Guangzhou Medical University.

### RNA Extraction and Quantitative Real-Time PCR Assays

Total RNAs were extracted and purified from human ccRCC samples using a Trizol kit (Invitrogen Corporation, Carlsbad, CA, United States) according to the manufacturer’s recommended protocol. cDNAs were synthesized by using the reverse transcription kit (Takara Biotechnology Dalian, China) with the following conditions: reverse transcription at 37°C for 15 min, followed by incubation at 85°C for 5 s in 20 μl of reaction volume. The reaction mixture for real-time PCR was prepared by following the manufacturer’s recommended protocols (Takara Clontech, Kyoto, Japan). Primer sequences of CEP55, IFI44, NCF4, T-cell immune regulator 1 (TCIRG1), and GAPDH are summarized in Supplementary Table S1. The cycle threshold (CT) values were standardized to CT values of GAPDH. The relative levels of individual mRNA in each sample transcript compared to control GAPDH were calculated using the 2^–ΔΔ^Ct method.

### Statistical Analysis

The 95% confidence interval (CI) and HR were generated by univariate and multivariate Cox proportional hazards regression analysis. The AUC of tROC curve and C-index were used to validate the prognostic performance of the mRNASig. The C-index and calibration plot were performed as an internal validation of the nomogram. The calibration plot was used to compare the predicted probability of OS versus actual OS at 5 years using the 200 bootstrap resamples to reduce overfitting bias. The Spearman correlation analysis was performed to assess the association between mRNA expression level and tumor microenvironment. Difference comparisons of two groups were conducted by Wilcoxon test. All the statistical analyses were performed in R version 3.6.2. and *P* < 0.05 was considered as statistically significant.

## Results

### Identification of Robust DEGs

**FIGURE 1:**
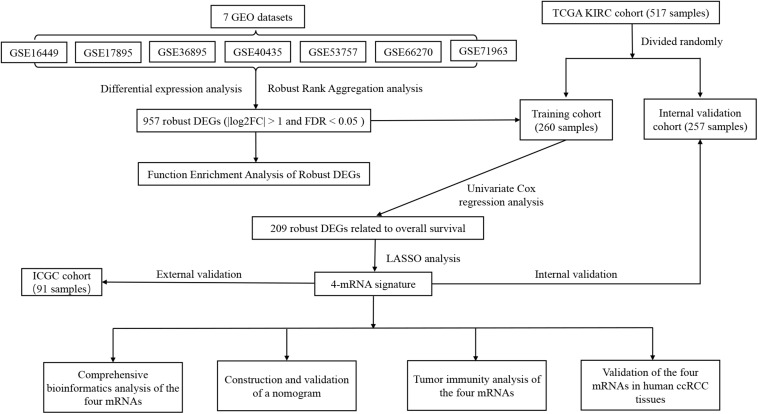
Flowchart showing the process of developing the mRNA signature and the nomogram of ccRCC in this study.

The flowchart of this study is shown in Figure 1. Table 1 shows the details of seven eligible GEO datasets (GSE16449, GSE17895, GSE36895, GSE40435, GSE53757, GSE66270, and GSE71963), which were identified as 5334, 6250, 4090, 7082, 4033, 3180, and 5314 DEGs, respectively. There were a total of 957 robust DEGs that included 445 up-regulated and 512 down-regulated mRNAs that were identified using RRA analysis. The top 20 up-regulated and down-regulated robust DEGs identified by RRA analysis are shown in the heat map (Supplementary Figure S1).

**TABLE 1:** Details of seven GEO datasets included in this study.

**Dataset ID**	**Sample size**		**Platform**
	**Normal**	**Tumor**	
GSE16449	52	18	Agilent-014850 Whole Human Genome Microarray 4x44K G4112F
GSE17895	22	138	Affymetrix GeneChip Human Genome U133 Plus 2.0 Array (MBNI v11 Entrez Gene ID CDF)
GSE36895	23	53	[HG-U133_Plus_2] Affymetrix Human Genome U133 Plus 2.0 Array
GSE40435	101	101	Illumina HumanHT-12 V4.0 expression beadchip
GSE53757	72	72	[HG-U133_Plus_2] Affymetrix Human Genome U133 Plus 2.0 Array
GSE66270	14	14	[HG-U133_Plus_2] Affymetrix Human Genome U133 Plus 2.0 Array
GSE71963	16	32	Agilent-014850 Whole Human Genome Microarray 4x44K G4112F

### Function Enrichment Analysis of Robust DEGs

The GO term has BP, CC, and MF as its categories. As shown in Supplementary Figure S2A, the GO term of BP including the small-molecule catabolic process, organic anion transport, carboxylic acid biosynthetic process, and nephron development for robust DEGs were detected. The CC with apical part of cell, apical plasma membrane, and extracellular matrix were significantly enriched. The significantly enriched MF included cofactor binding, active transmembrane transporter activity, and coenzyme binding. According to the KEGG pathway analysis (Supplementary Figure S2B), the phagosome, carbon metabolism, and cell adhesion molecules (CAMs) were mostly associated with the robust DEGs.

### Survival Analysis of Robust DEGs and Development of mRNASig

Through the univariate Cox regression analysis, a total of 209 robust DEGs were identified to be closely associated with OS, which included 91 up-regulated DEGs with HR > 1 and 118 down-regulated DEGs with HR < 1. Later, these 209 survival-related DEGs were included into LASSO analysis with 10-fold cross-validation (Supplementary Figure S3). The mRNASig consisting of mRNASig that included CEP55, IFI44, NCF4, and TCIRG1 was developed according to the LASSO coefficient and the relative expression levels of the mRNAs. The up-regulated CEP55, IFI44, NCF4, and TCIRG1 with HR > 1 were regarded as oncogenes. The formula of risk score was as follows: Risk score = (CEP55 × 0.248 + IFI44 × 0.259 + NCF4 × 0.196 + TCIRG1 × 0.37).

**FIGURE 2:**
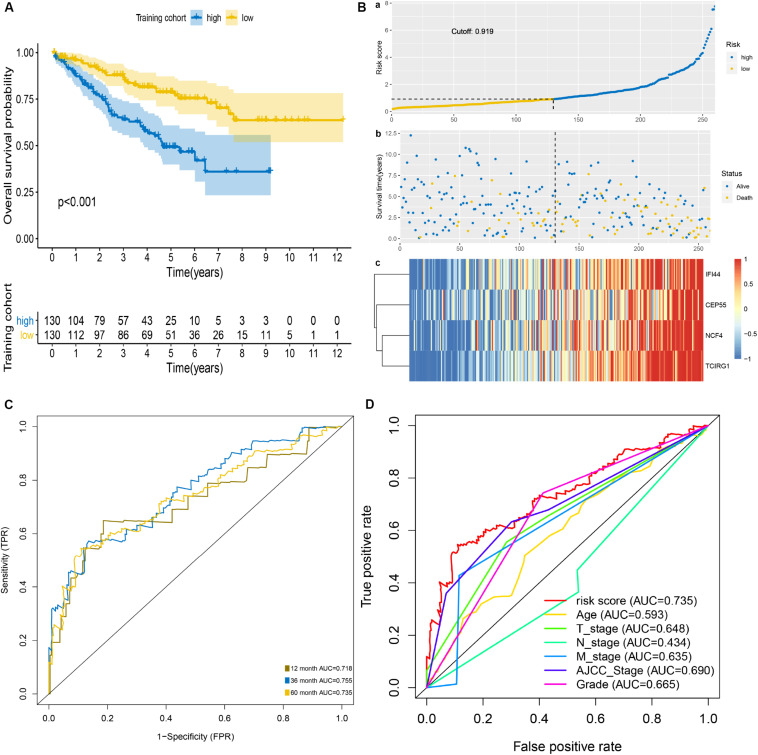
Prognostic analysis of mRNASig in the training cohort. **(A)** Kaplan–Meier analysis of mRNASig. **(B)** The distribution of risk score **(a)**, survival status **(b)**, and mRNAs expression levels of patients **(c)** in training cohort. **(C)** Time-dependent ROC analysis of mRNASig. **(D)** Comparison of the predictive power among risk score, age, AJCC-stage, T-stage, N-stage, M-stage, and grade.

The patients of the training cohort were stratified into low- and high-risk groups based on the cutoff value of 0.919. The Kaplan–Meier analysis revealed that the group with higher risk scores had significantly unfavorable OS (*P* < 0.001) (Figure 2A). The distribution of risk score, survival status, and mRNA expression levels of patients in training cohort is shown in Figure 2B. In addition, the mRNASig had a good predictive performance with AUCs of 0.718, 0.755, and 0.735 at 1, 3, and 5 years, respectively (Figure 2C), with C-index of 0.70 (95% CI, 0.62–0.79). The AUC value of 5 years of the mRNASig was higher than clinical variables, which suggested that the mRNASig may be a better predictor of OS of ccRCC patients (Figure 2D).

### Internal and External Validation of mRNASig

**FIGURE 3:**
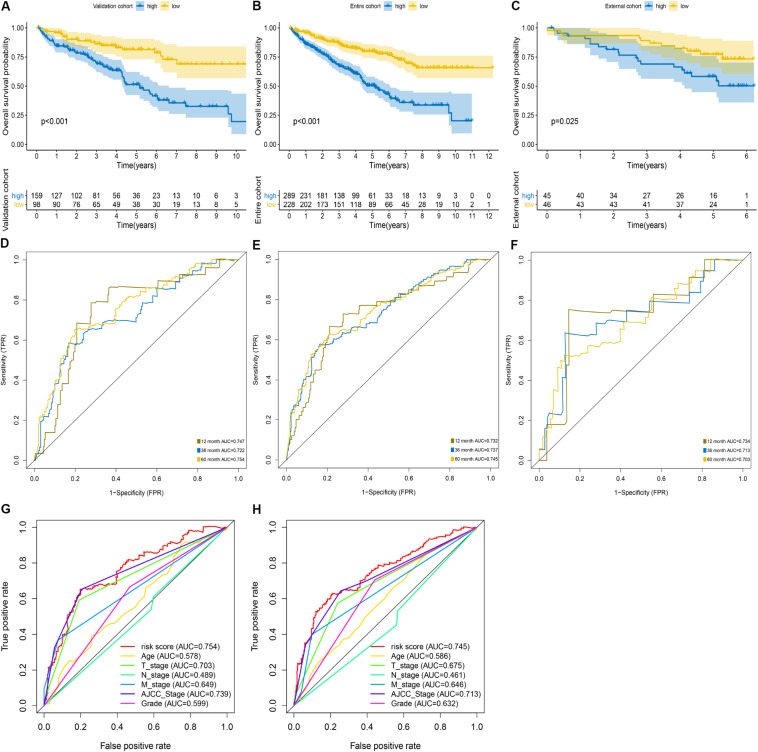
Validation of mRNASig. ICGC cohort was regarded as the external validation set. Kaplan–Meier analysis of mRNASig in internal validation cohort **(A)**, the entire cohort **(B)**, and external validation cohort **(C)**. Time-dependent ROC analysis of mRNASig in internal validation cohort **(D)**, the entire cohort **(E)**, and external validation cohort **(F)**. Comparison of the predictive power among risk score, age, AJCC-stage, T-stage, N-stage, M-stage, and grade in internal validation cohort **(G)** and the entire cohort **(H)**.

We used the internal validation cohort, the entire cohort, and the external validation cohort to assess the mRNASig constructed in the training cohort. Being consistent with the result of the training cohort, the Kaplan–Meier analysis of the three validation cohorts revealed that the prognosis of ccRCC patients was worse in the high-risk group than in the low-risk group (*P* < 0.001; Figures 3A–C). The mRNASig also had an excellent predictive performance in the internal validation cohort with AUC of 0.747, 0.722, and 0.754 at 1, 3, and 5 years, and with C-index of 0.70 (95% CI, 0.61–0.78) (Figure 3D). AUC for 1-, 3-, and 5-year OS of the entire cohort was 0.732, 0.737, and 0.745, and C-index was 0.70 (95% CI, 0.62–0.80) (Figure 3E). Similarly, the AUC for 1-, 3-, and 5-year OS of the external validation cohort was 0.734, 0.713, and 0.703, respectively, with a C-index of 0.68 (95% CI, 0.60–0.77) (Figure 3F). The AUC value of 5 years of the mRNASig was higher than clinical variables in the internal validation cohort and the entire cohort (Figures 3G,H).

### Identification of Independent Prognostic Variables in ccRCC

The univariate Cox proportional hazards regression analysis revealed that the risk score, age, AJCC stage, grade, T stage, N stage, and M stage were significantly associated with OS of ccRCC in the training cohort (Table 2), the internal validation cohort (Supplementary Table S2), and the entire cohort (Supplementary Table S3). Moreover, the multivariate Cox proportional hazards regression analysis revealed that risk score, age, AJCC stage, and grade were independent prognostic factors of OS. Subsequently, we used the Kaplan–Meier analysis to investigate the predictive value of the mRNASig in different subgroups stratified by age, AJCC stage, grade, and T stage of the entire cohort. The patients of the high-risk groups had an unfavorable OS compared to the patients of low-risk groups in ≤60 years, >60 years, T1 and T2, T3 and T4, AJCC stage I and II, AJCC stage III and IV, Grade 1 and 2, and Grade 3 and 4 subgroups (Supplementary Figures S4A–H).

**TABLE 2:** Cox regression analysis of mRNASig and OS of ccRCC in the training cohort.

**Variables**	**Univariate analysis**		**Multivariate analysis**	
	
	**HR (95% CI)**	***P***	**HR (95% CI)**	***P***
Age	1.08 (1.01–1.05)	0.002	1.04 (1.01–1.06)	0.005
T stage				
T1	Ref			
T2	1.53 (0.74–3.14)	0.2		
T3	2.7 (1.61–4.45)	<0.001		
T4	9.79 (3.70–25.89)	<0.001		
N Stage				
N0	Ref			
N1	5.72 (2.42–13.52)	<0.001		
NX	0.69 (0.43–1.10)	0.2		
M Stage				
M0	Ref			
M1	4.67 (2.98–7.29)	<0.001		
MX	0.56 (0.076–4.08)	0.6		
AJCC Stage				
Stage I	Ref			
Stage II	0.90 (0.34–2.41)	0.8	0.85 (0.31–2.27)	0.7
Stage III	2.0 (1.10–3.65)	0.02	1.22 (0.65–2.28)	0.5
Stage IV	6.28 (3.64–10.84)	<0.001	4.32 (2.43–7.70)	<0.001
Grade				
G1 and G2	Ref			
G3 and G4	2.94 (1.79–4.84)	<0.001	2.24 (1.33–3.77)	0.002
Risk score				
Low	Ref			
High	2.73 (1.71–4.35)	<0.001	2.49 (1.52–4.05)	<0.001

### Construction and Validation of a Nomogram

**FIGURE 4:**
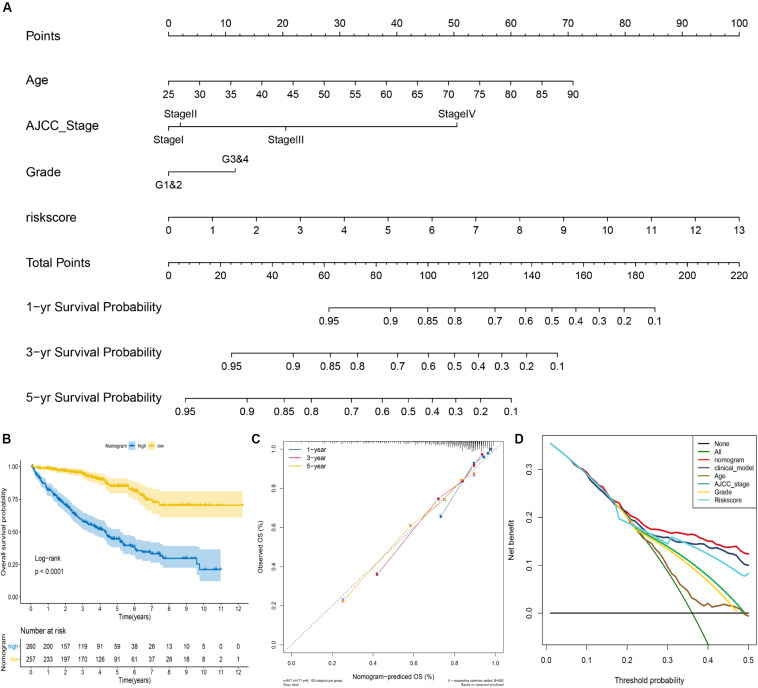
Construction of the nomogram for predicting overall survival of ccRCC in the entire cohort. **(A)** The nomogram predicting 1-, 3-, and 5-year overall survival of ccRCC. **(B)** Kaplan–Meier analysis of the nomogram. **(C)** The calibration plot for internal validation of the nomogram. **(D)** Decision curve analysis for 5-year overall survival prediction shows that the nomogram had the highest net benefit across 0–50% threshold probabilities.

To facilitate the clinical decision making, we constructed a nomogram based on the entire cohort to predict the probability of 1-, 3-, and 5-year OS of ccRCC patients. The risk score, age, AJCC stage, and grade were also included in the nomogram by a stepwise Cox regression model (Figure 4A). Kaplan–Meier analysis revealed that group with higher risk scores had significantly unfavorable OS (*P* < 0.001) (Figure 4B). In addition, we constructed a clinical model based on age, AJCC stage, and grade using a stepwise Cox regression model. The C-index of the nomogram was 0.78 (95% CI, 0.75–0.82), while that for the clinical model was 0.76 (95% CI, 0.67–0.85) and the risk score was 0.70 (95% CI, 0.61–0.79). The calibration plots showed that the prediction probability of nomogram is consistent with actual probability of OS at 5 years (Figure 4C). Moreover, the DCA for 5-year survival probability prediction shows that nomogram had the highest net benefit across 0–50% threshold probabilities (Figure 4D). Meanwhile, the net benefit of mRNASig was higher than age, AJCC stage, and grade.

### Comprehensive Bioinformatics Analysis of mRNASig mRNAs

**FIGURE 5:**
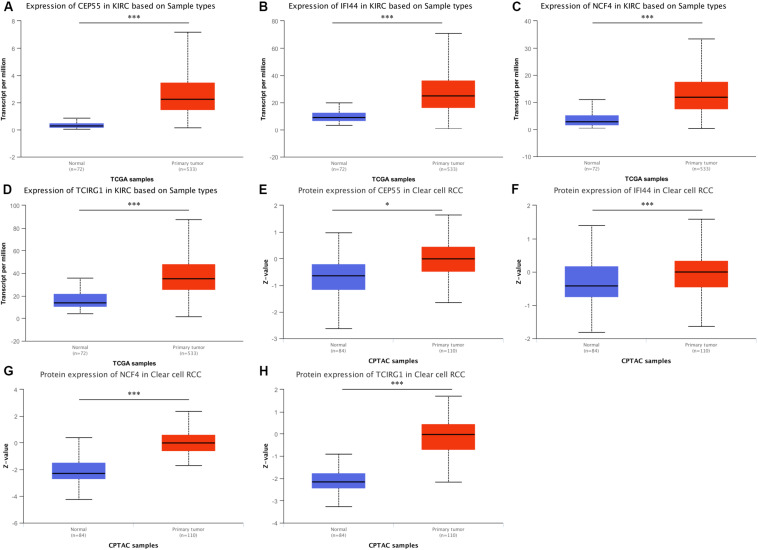
**(A–D)** Expression pattern of CEP55, IFI44, NCF4, and TCIRG1 between ccRCC and adjacent normal tissue. **(E–H)** Corresponding protein level between ccRCC and adjacent normal tissue. *P < 0.05 and ***P < 0.001 respectively.

According to the results of the UALCAN website, the mRNA expression levels of CEP55, IFI44, NCF4, and TCIRG1 were significantly up-regulated in ccRCC tissues compared to non-tumorous tissues (Figures 5A–D). The protein level of mRNASig mRNAs was detected by the UALCAN website, and the results were similar to the mRNA expression levels in ccRCC tissues (Figures 5E–H). We explored the association between the mRNASig and clinical traits including overall survival analysis, cancer stage, and tumor grade on the TISIDB website (Supplementary Figure S5). As shown in Supplementary Figure S5, the four up-regulated mRNAs did not only have poorer prognosis than down-regulated mRNAs but correlated positively as well with higher AJCC stage and tumor grade, suggesting an important contribution to the pathogenesis of ccRCC. Furthermore, the Kaplan–Meier analysis of the mRNASig was performed in the external cohort (the ICGC cohort) and showed that the high expression of mRNAs had worse OS compared with the low expression mRNAs (Supplementary Figure S5).

The ROC curve analysis was performed to assess the sensitivity and specificity of mRNASig mRNAs and the corresponding proteins for the diagnosis of ccRCC in the TCGA database. The ROC curves of CEP55, IFI44, NCF4, and TCIRG1 in the TCGA database are displayed and show good efficacy in diagnosing ccRCC with AUC of 0.934, 0.881, 0.924, and 0.913 respectively (Supplementary Figure S6A). Moreover, we found that the corresponding proteins also have good efficacy in diagnosing ccRCC with AUC of 0.725, 0.685, 0.929, and 0.975, respectively (Supplementary Figure S6B).

**FIGURE 6:**
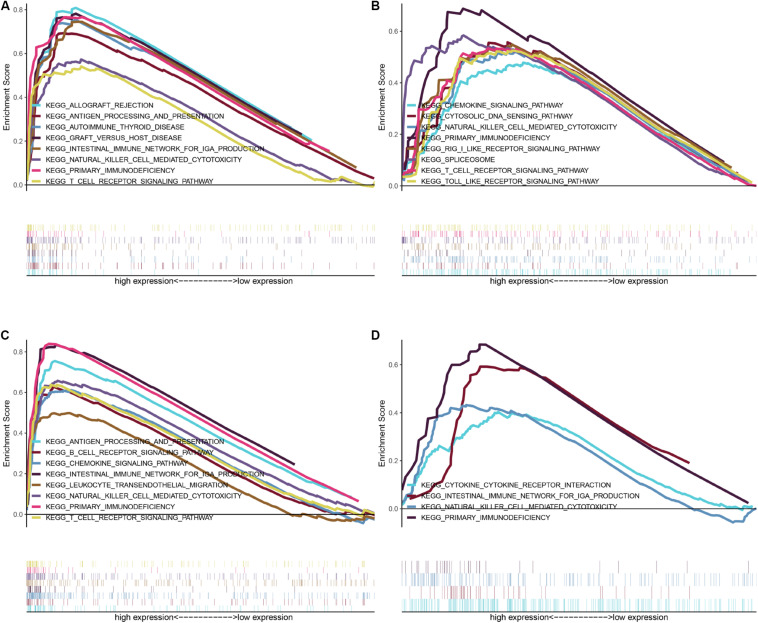
GSEA of KEGG pathway gene sets in CEP55 **(A)**, IFI44 **(B)**, NCF4 **(C)**, and TCIRG1 **(D)** high versus low samples from TCGA database. Normalized enrichment score (NES) is shown in each plot.

To further investigate the potential molecular mechanisms of CEP55, IFI44, NCF4, and TCIRG1 in ccRCC, we performed GSEA using TCGA ccRCC RNA-seq data. As shown in Figure 6, genes in high-expression groups of CEP55, IFI44, NCF4, and TCIRG1 were all enriched with “Natural killer cell mediated cytotoxicity” and “Primary immunodeficiency” pathways; the “T cell receptor signaling pathway” was enriched in high-expression groups of CEP55, IFI44, and NCF4, whereas the “Intestinal immune network for IgA production” pathway was enriched in CEP55, NCF4, and TCIRG1 high-expression groups, respectively. The results of GSEA revealed that CEP55, IFI44, NCF4, and TCIRG1 were all closely associated with immune signaling pathways.

### Validation of mRNASig mRNAs in Human ccRCC Tissues

**FIGURE 7:**
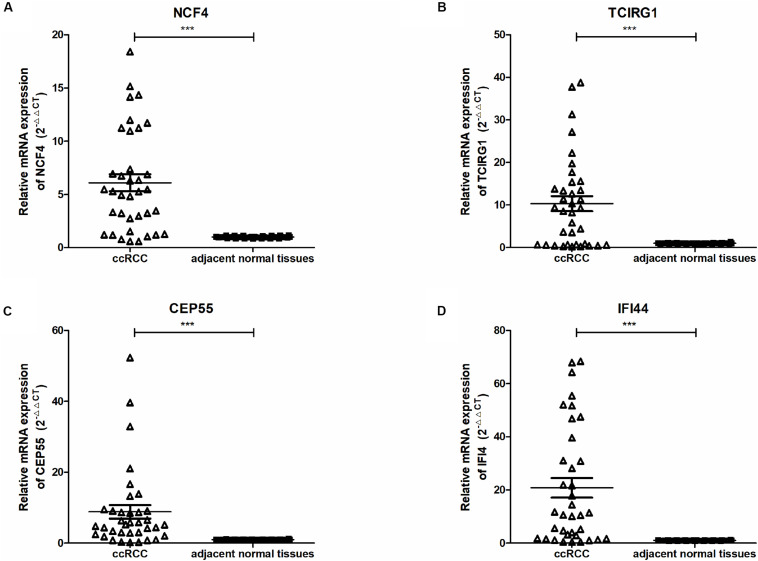
**(A–D)** Expression of NCF4, TCIRG1, CEP55, and IFI44, between ccRCC and adjacent normal tissues were detected by qRT-PCR. ***P < 0.001.

To further confirm the correlation of mRNASig mRNAs expression with tumor progression in ccRCC, we conducted quantitative real-time PCR (qRT-PCR) assays in human tissue samples. The clinicopathological characteristics of 36 patients are presented in Supplementary Table S4. As shown in Figure 7, CEP55, IFI44, NCF4, and TCIRG1 mRNA expression were found to be remarkedly higher in ccRCC tissues than those in adjacent normal renal tissues (*n* = 36, *P* < 0.001). This result implied that these mRNASig mRNAs were increased significantly in most of ccRCC tissues and might be used as valuable biomarkers for ccRCC patients.

### Tumor Immunity Analysis of mRNASig mRNAs

The CEP55, IFI44, NCF4, and TCIRG1 participated in a variety of immune signaling pathways as per the results of GSEA. Moreover, the immune environment was closely related to the development of tumor and the effect of immunotherapy. Therefore, we used the TIMER website to further explore the relationship between mRNASig mRNAs and TILs. As shown in Supplementary Figure S7, the mRNA expression levels of CEP55, IFI44, NCF4, and TCIRG1 were positively correlated with TILs, including B cells, CD4+ T cells, CD8+ T cells, neutrophils, macrophages, and dendritic cells. These results revealed that mRNASig mRNAs play a critical role in immune infiltration in ccRCC.

**FIGURE 8:**
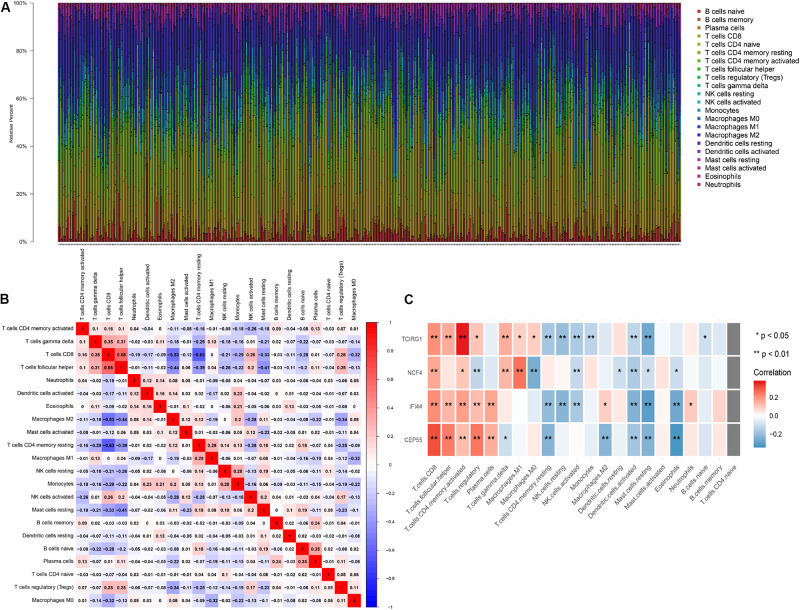
The landscape of immune infiltration in TCGA ccRCC patients. **(A)** The abundance fraction of 22 tumor-infiltrating lymphocytes (TILs) in the 396 ccRCC samples. Each column represents a sample, and each column with a different color and height indicates the abundance fraction of TILs in this sample. **(B)** The correlation between the abundance fraction of various immune cells. The value represents the correlation value. Red represents a positive correlation, and blue represents a negative correlation. **(C)** The relationship between expression of CEP55, IFI44, NCF4, and TCIRG1 and 22 TILs.

The 22 TILs were measured on the CIBERSORT website, and the correlation between them and mRNASig mRNAs was validated using the Spearman correlation analysis. The abundance fraction of 22 TILs in the 396 ccRCC samples was different (Figure 8A). Therefore, the difference in the proportion of TILs between individuals might represent tumor heterogeneity. In addition, the correlation between the proportion of different TIL subpopulations ranged from weak to moderate (Figure 8B). The Spearman correlation analysis results between the mRNASig mRNAs and 22 TILs are shown in Figure 8C and revealed that mRNASig mRNAs were closely related to a variety of TILs, especially including CD8+ T cells, activated CD4+ memory T cell, regulatory T cell, follicular helper T cells, and activated NK cells.

**FIGURE 9:**
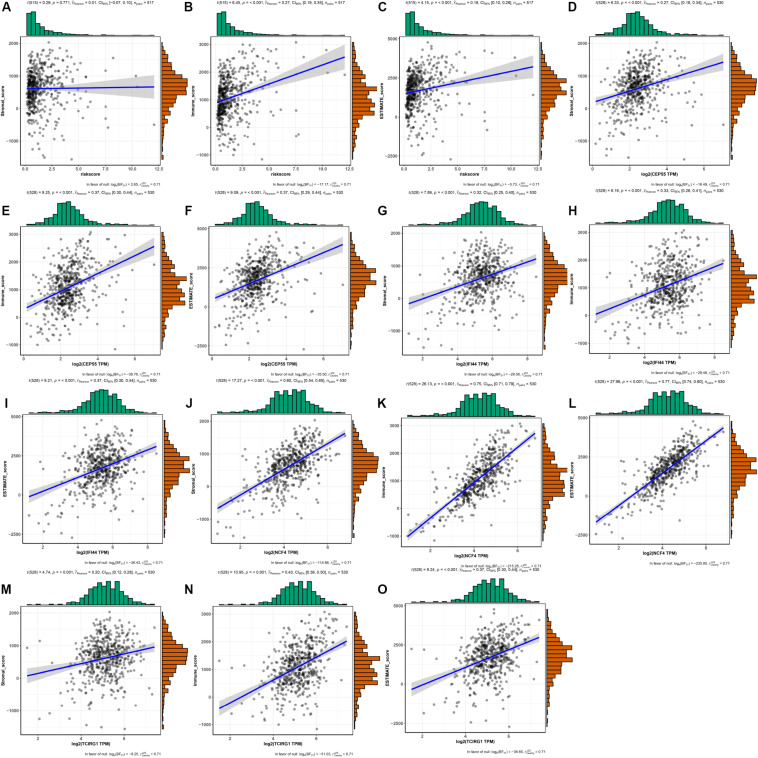
The associations between risk score and the expression of the mRNASig (CEP55, IFI44, NCF4, and TCIRG1) and TME score (stromal, immune, and estimate scores). **(A–C)** Risk score. **(D–F)** CEP55. **(G–I)** IFI44. **(J–L)** NCF4. **(M–O)** TCIRG1.

**FIGURE 10:**
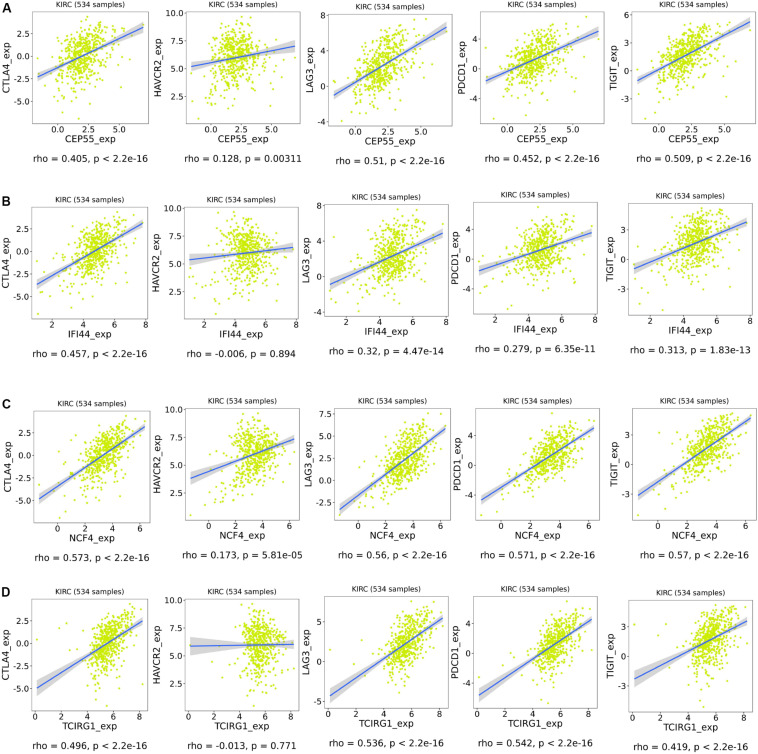
Association of expression of CEP55 **(A)**, IFI44 **(B)**, NCF4 **(C)**, and TCIRG1 **(D)** with the expression of CTLA4, HAVCR2, LAG3, PDCD1, and TIGIT in ccRCC.

As shown in Figure 9, the risk score and the expression of the mRNASig were positively correlated with immune, stromal, and estimate scores. These results suggested that mRNASig mRNAs may serve as biomarkers and targets for immunotherapy and revealed that the high-risk group was more sensitive to the treatment of immunotherapy. Moreover, we found that mRNASig mRNAs were positively correlated with the expression of CTLA4, LAG3, PDCD1, TIGIT, and HAVCR2, displaying that the poor prognosis of up-regulated mRNASig mRNAs may be related to the immunosuppressive microenvironment (Figure 10).

## Discussion

The ccRCC is the most common subtype of RCC and is a highly malignant tumor with insidious onset, high recurrence rate, and high mortality (Hsieh et al., 2017). At present, the traditional TNM staging system is commonly used as a prognosis indicator of ccRCC patients but is not precise enough to identify the high-risk ccRCC patients who need more radical treatment (Veeratterapillay et al., 2012). Therefore, it is necessary to develop novel signatures to predict the prognosis of renal cancer.

In our study, we identified 957 robust DEGs of ccRCC by the RRA method of seven GEO datasets. The GO and KEGG analysis revealed that the robust DEGs are closely associated with pathogenesis of ccRCC. The Univariate Cox regression analysis identified the 209 DEGs associated with OS of ccRCC patients. We used the LASSO analysis to establish the mRNASig, consisting of CEP55, IFI44, NCF4, and TCIRG1. The mRNASig had a good predictive accuracy and was validated by the internal cohort and the external cohort. The patients in high-risk groups had significantly poorer prognosis compared to those in low-risk groups. Meanwhile, it is an independent prognostic factor of ccRCC, which can greatly improve the predictive ability of clinical models. A nomogram combined with mRNASig and clinicopathological variables is constructed to predict OS and to assist clinicians in making personalized treatment decisions. Although previous studies have developed several signatures to identify high-risk ccRCC patients, these studies did not have enough samples and needed more independent validation (Qu et al., 2018; Wei et al., 2019). Furthermore, mRNASig had a better prognostic performance than the other reported signature (RCClnc4 signature and six-SNP-based classifier) for ccRCC.

The up-regulated CEP55, IFI44, NCF4, and TCIRG1 with HR > 1 were regarded as oncogenes and were proved to be closely related to tumor progression by using comprehensive bioinformatics analysis. Moreover, we found that the proteins of CEP55, IFI44, NCF4, and TCIRG1 are also up-regulated in ccRCC tissues. It is revealed that protein from these may also play a key role in ccRCC. The study of Chen et al. (2019c) had shown that up-regulated CEP55 could promote epithelial-to-mesenchymal transition, migration, and invasion through the PI3K/AKT/mTOR pathway in RCC. Similarly, CEP55 was found to be up-regulated in multiple types of cancer and was related to unfavorable prognosis of non-small-cell lung cancer patients (Jiang et al., 2018) and breast cancer patients (Yin et al., 2018). The IFI44 is one of the interferon-α-stimulated genes that is associated with hepatitis D (DeDiego et al., 2019) and limited scleroderma (Bodewes et al., 2018). In addition, the down-regulated IFI44 in lymphocytes of breast cancer leads to immune dysfunction (Critchley-Thorne et al., 2009). The NCF4, an innate immunity gene, is the part of the NAPDH complex and is associated with an increased susceptibility to Crohn’s disease (Roberts et al., 2008). There is a provided evidence of an association between NCF4 and increased risk of colorectal cancer (Ryan et al., 2014), whereas the NCF4 rs1883112 were risk factors in diffuse large B-cell lymphoma patients (Liu et al., 2017). The TCIRG1 was up-regulated in hepatocellular carcinoma and significantly associated with unfavorable prognosis of hepatocellular carcinoma patients (Yang et al., 2018). However, there has been no study of IFI44, NCF4, and TCIRG1 in ccRCC, and the MFs of those are worth exploring further.

Clear cell renal cell carcinoma has been proven to be a hot tumor (highly immune-infiltrated), and TME plays an important role in the control or progression of ccRCC (Galon and Bruni, 2019). Currently, immunotherapy is an option of systemic treatment for metastatic ccRCC (Rini et al., 2016) and had great potential. Moreover, GSEA revealed that CEP55, IFI44, NCF4, and TCIRG1 were involved in multiple immune signaling pathways. We found that CEP55, IFI44, NCF4, and TCIRG1 were positively correlated with multiple TILs, especially including CD8+ T cells, activated CD4+ memory T cells, and Tregs. The previous studies found that increased CD4+ and CD8+ T cells were associated with tumor progression and poor prognosis in RCC (Nakano et al., 2001; Remark et al., 2013). Multiple studies have shown that high numbers of Tregs could suppress the anti-tumor immune response and was associated with poor prognosis of RCC (Liotta et al., 2011; Kang et al., 2013; Polimeno et al., 2013). In addition, mRNASig mRNAs were negatively correlated with activated NK cells and resting NK cells. Low NK-cell densities were associated with poorer survival in RCC (Remark et al., 2013). These results suggest that CEP55, IFI44, NCF4, and TCIRG1 may promote tumor progression by regulating TILs in ccRCC. It is of great significance to further explore the specific mechanisms by which CEP55, IFI44, NCF4, and TCIRG1 regulate these TILs in ccRCC.

CTLA4 can inhibit the early activation and differentiation of T cells, while PDCD1 regulates T cell effector function, which may result in its exhaustion (Buchbinder and Desai, 2016; Hellmann et al., 2016). LAG3, a co-inhibitory receptor on TILs, enhances activity of Tregs and modulates proliferation, differentiation, and effector function of TILs (Hellmann et al., 2016). TIGIT (Manieri et al., 2017) and HAVCR2 (a marker for exhausted TILs) (Du et al., 2017) are possible targets for binding to existing ICIs. In our study, we found that mRNASig mRNAs were positively correlated with the expression of the above immune checkpoint inhibitors, showing that the poor prognosis of up-regulated mRNASig mRNAs may be related to the immunosuppressive microenvironment and mRNASig mRNAs may be novel potential targets for future treatment of ccRCC.

Our study provides new insights into TME and immune-related therapy of ccRCC. However, there are some limitations to this study. First, there exists heterogeneity in this retrospective study, and more prospective studies are needed to validate this. Second, the molecular mechanisms of CEP55, IFI44, NCF4, and TCIRG1 found in this study require further study.

## Conclusion

In summary, we developed and validated mRNASig that is based on mRNASig and has independent prognostic significance for ccRCC patients. A nomogram was constructed combining mRNASig, AJCC stage, and grade to accurately identify high-risk patients who need more radical treatment. Furthermore, we found that CEP55, IFI44, NCF4, and TCIRG1 contributed to poor prognosis for ccRCC and played an important role in the TME of ccRCC through regulating TILs or ICIs. It suggests that CEP55, IFI44, NCF4, and TCIRG1 may be novel potential targets for future immunotherapy of ccRCC. Notably, our study provided new insights for researchers to explore the treatment of ccRCC.

## Data Availability Statement

All datasets presented in this study are included in the article/Supplementary Material.

## Author Contributions

XW and ZZ performed the data analyses. CC and DL assisted in collecting the data. YL and DL obtained the funding. XW and ZZ wrote the manuscript. DG and AK commented and revised the manuscript. YL and DG supervised the study. All authors participated in preparing the manuscript and approved the final submitted and published version.

## Conflict of Interest

The authors declare that the research was conducted in the absence of any commercial or financial relationships that could be construed as a potential conflict of interest.

## Footnotes

1 https://www.ncbi.nlm.nih.gov/geo/

2 https://cancergenome.nih.gov/

3 https://icgc.org/

4 http://ualcan.path.uab.edu/analysis.html

5 http://cis.hku.hk/TISIDB/index.php

6 https://proteomics.cancer.gov/programs/cptac

7 https://cistrome.shinyapps.io/timer/

8 https://cibersort.stanford.edu/

9 https://bioinformatics.mdanderson.org/estimate/index.html

## Supplementary Material

FIGURE S1 Identification of robust DEGs by RRA method. Heatmap presenting the top 20 upregulated (Red) and top 20 downregulated (Green) mRNAs according to *P-*value. The numbers in the heatmap represent log2-fold change in each dataset calculated by the “limma” R package. DEG, differentially expressed gene; RRA, robust rank aggregation.

FIGURE S2 GO and KEGG analysis of the robust DEGs. **(A)** Top 10 of GO terms of the robust DEGs, including biological process, cellular component, molecular function. **(B)** Top 30 enriched KEGG pathways of the robust DEGs.

FIGURE S3 **(A)** LASSO coefficients profiles of 209 mRNAs. **(B)** LASSO regression with 10-fold cross-validation obtained 4 prognostic mRNAs that error is within 1 standard error of the minimum.

FIGURE S4 Kaplan–Meier analysis of mRNASig risk score level in different subgroups including ≤60 years **(A)**, >60 years **(B)**, T1 and T2 **(C)**, T3 and T4 **(D)**, AJCC stage I and II **(E)**, AJCC stage III and IV **(F)**, Grade 1 and 2 **(G)**, and Grade 3 and 4 **(H)**.

FIGURE S5 Validation of CEP55, IFI44, NCF4, and TCIRG1 in the TCGA ccRCC database and the ICGC cohort. **(A–D)** Expression of CEP55 in ccRCC samples with different AJCC-stages, tumor grade and overall survival. **(E–H)** Expression of IFI44 in ccRCC samples with different AJCC-stages, tumor grade and overall survival. **(I–L)** Expression of NCF4 in ccRCC samples with different AJCC-stages, tumor grade and overall survival. **(M-P)** Expression of TCRIG1 in ccRCC samples with different AJCC-stages, tumor grade, and overall survival.

FIGURE S6 ROC curve analysis of CEP55, IFI44, NCF4, and TCIRG1 **(A)** and corresponding proteins **(B)** for the diagnosis of ccRCC in TCGA database.

FIGURE S7 Association of expression of CEP55 **(A)**, IFI44 **(B)**, NCF4 **(C)**, and TCIRG1 **(D)** with six tumor-infiltrating lymphocytes in ccRCC. *P* < 0.05 is regarded as statistically significant. Each dot represents a ccRCC sample in the TCGA database.
