# Supplementary figures and images for: Identification of a Novel Signature and Construction of a Nomogram Predicting Overall Survival in Clear Cell Renal Cell Carcinoma
Source: Front Genet. 2020 Sep 4;11:1017. doi: 10.3389/fgene.2020.01017 (PMC7500318; doi:10.3389/fgene.2020.01017)

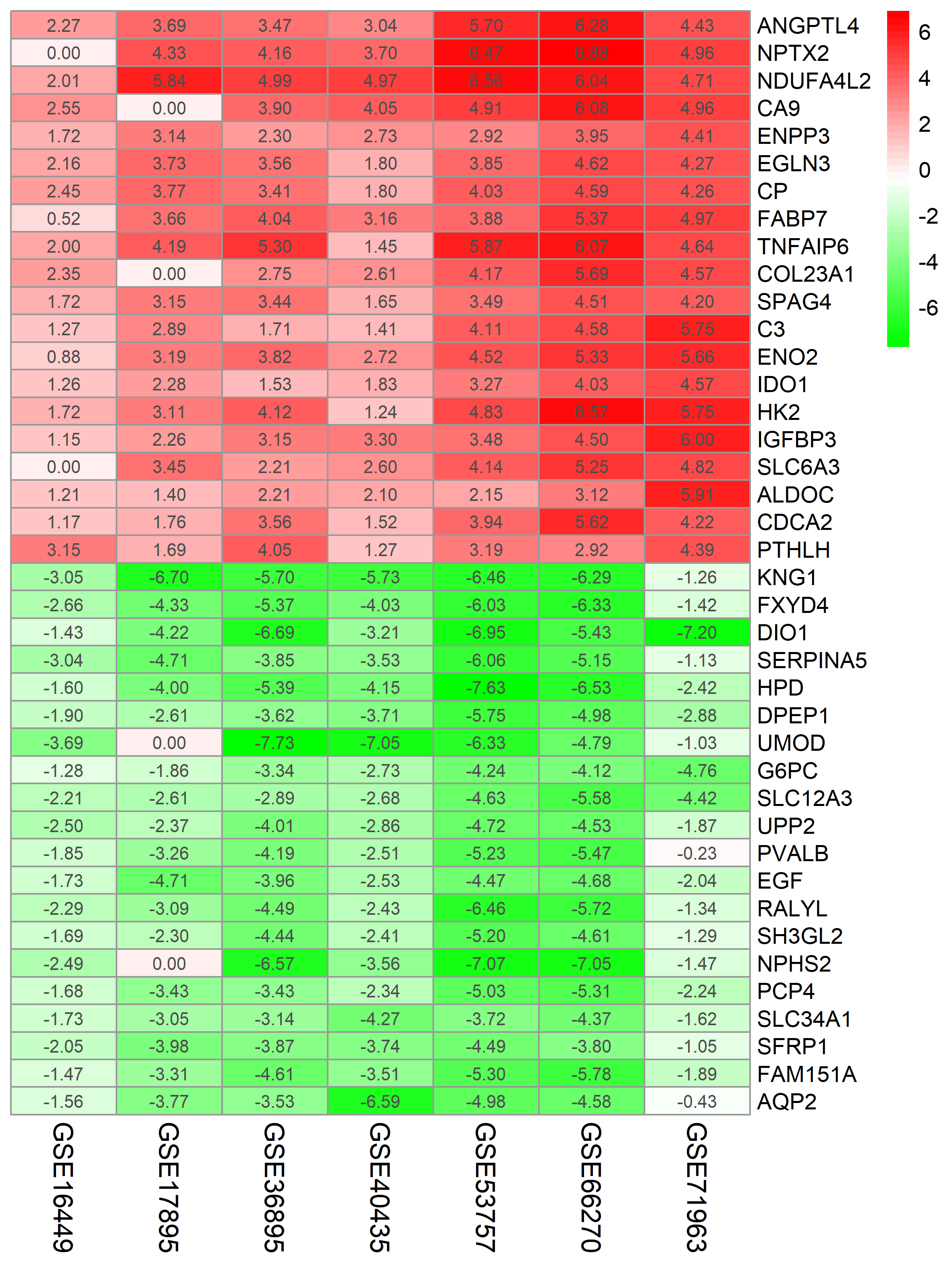

Supplement: FIGURE S1 — Identification of robust DEGs by RRA method. Heatmap presenting the top 20 upregulated (Red) and top 20 downregulated (Green) mRNAs according to P-value. The numbers in the heatmap represent log2-fold change in each dataset calculated by the “limma” R package. DEG, differentially expressed gene; RRA, robust rank aggregation. [file Image_1.TIFF]

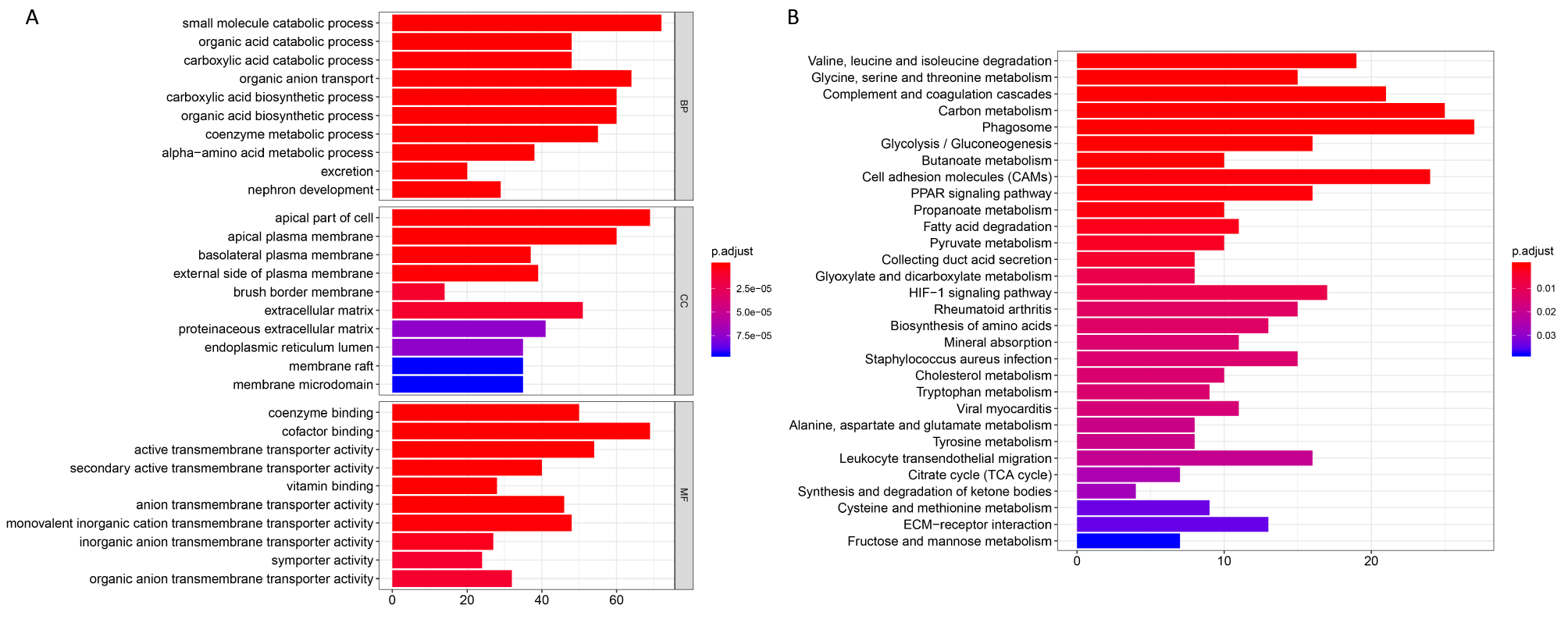

Supplement: FIGURE S2 — GO and KEGG analysis of the robust DEGs. (A) Top 10 of GO terms of the robust DEGs, including biological process, cellular component, molecular function. (B) Top 30 enriched KEGG pathways of the robust DEGs. [file Image_2.TIF]

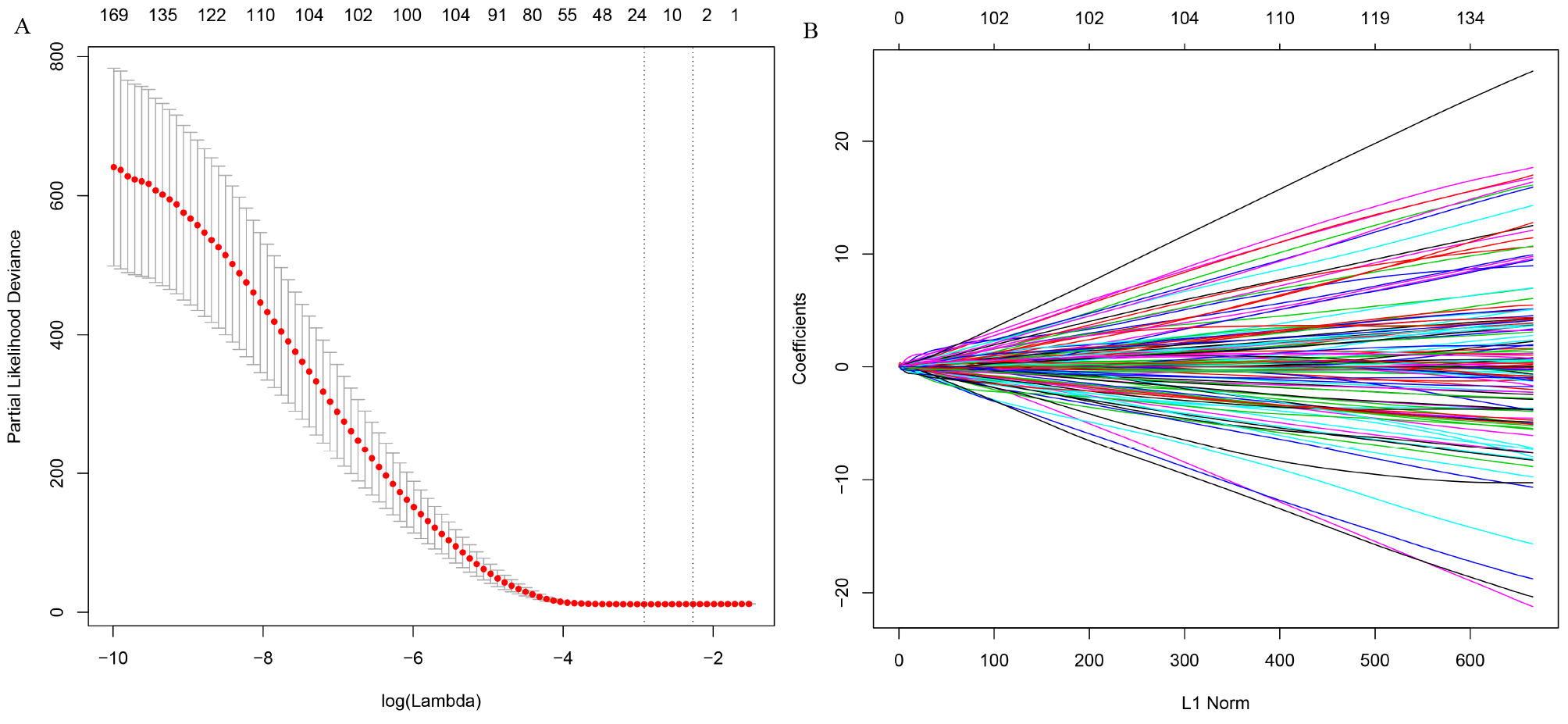

Supplement: FIGURE S3 — (A) LASSO coefficients profiles of 209 mRNAs. (B) LASSO regression with 10-fold cross-validation obtained 4 prognostic mRNAs that error is within 1 standard error of the minimum. [file Image_3.TIF]

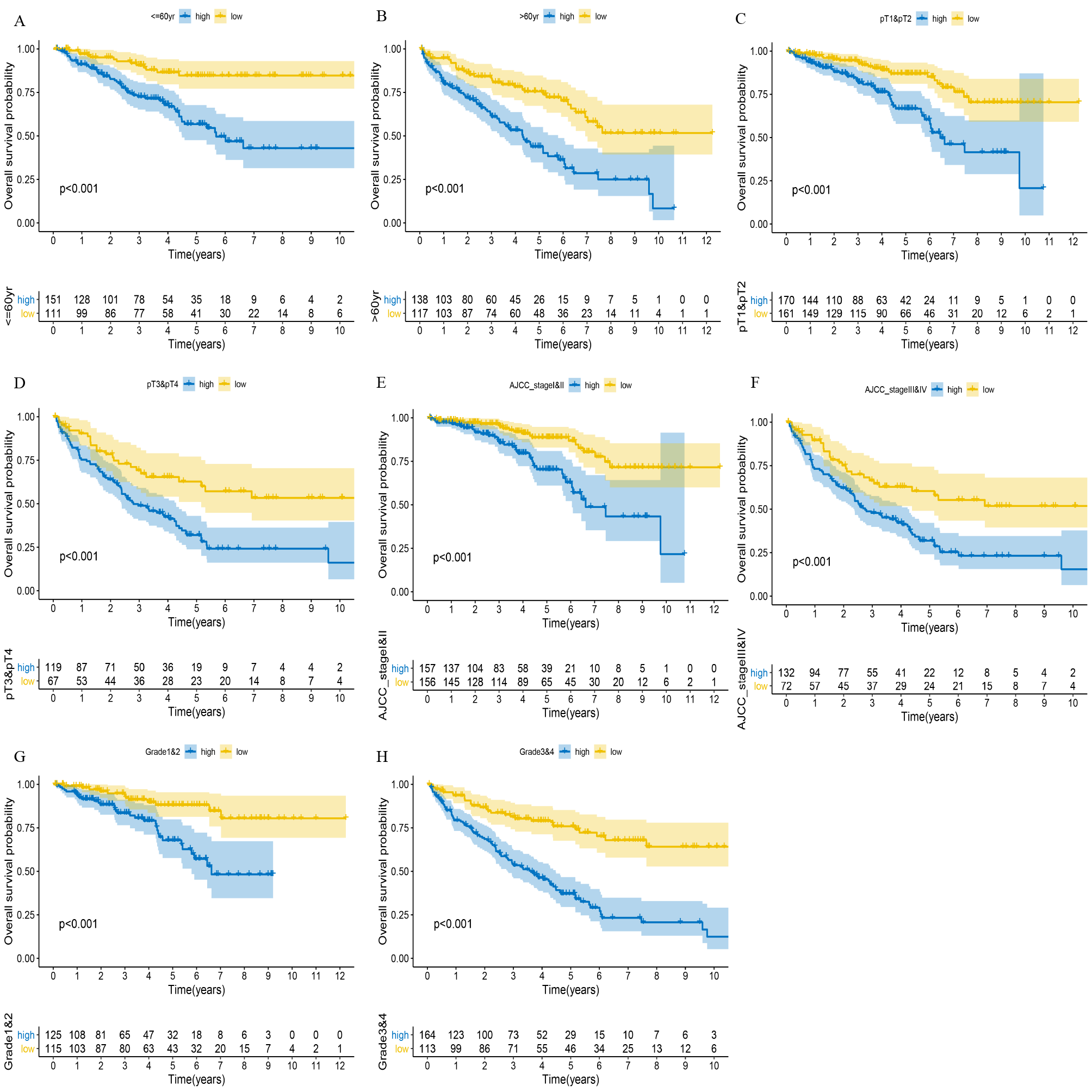

Supplement: FIGURE S4 — Kaplan–Meier analysis of mRNASig risk score level in different subgroups including ≤60 years (A), >60 years (B), T1 and T2 (C), T3 and T4 (D), AJCC stage I and II (E), AJCC stage III and IV (F), Grade 1 and 2 (G), and Grade 3 and 4 (H). [file Image_4.TIF]

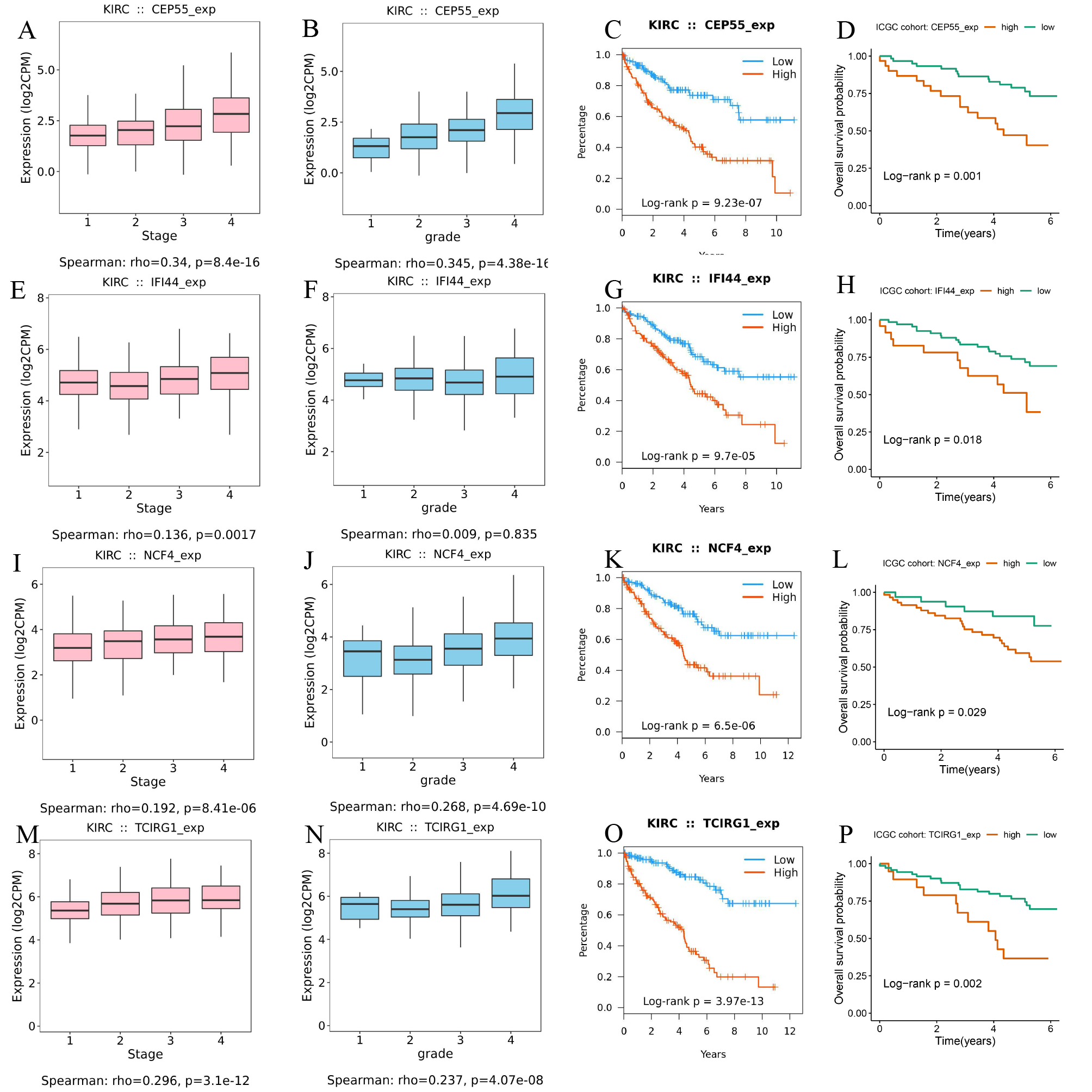

Supplement: FIGURE S5 — Validation of CEP55, IFI44, NCF4, and TCIRG1 in the TCGA ccRCC database and the ICGC cohort. (A–D) Expression of CEP55 in ccRCC samples with different AJCC-stages, tumor grade and overall survival. (E–H) Expression of IFI44 in ccRCC samples with different AJCC-stages, tumor grade and overall survival. (I–L) Expression of NCF4 in ccRCC samples with different AJCC-stages, tumor grade and overall survival. (M-P) Expression of TCRIG1 in ccRCC samples with different AJCC-stages, tumor grade, and overall survival. [file Image_5.TIF]

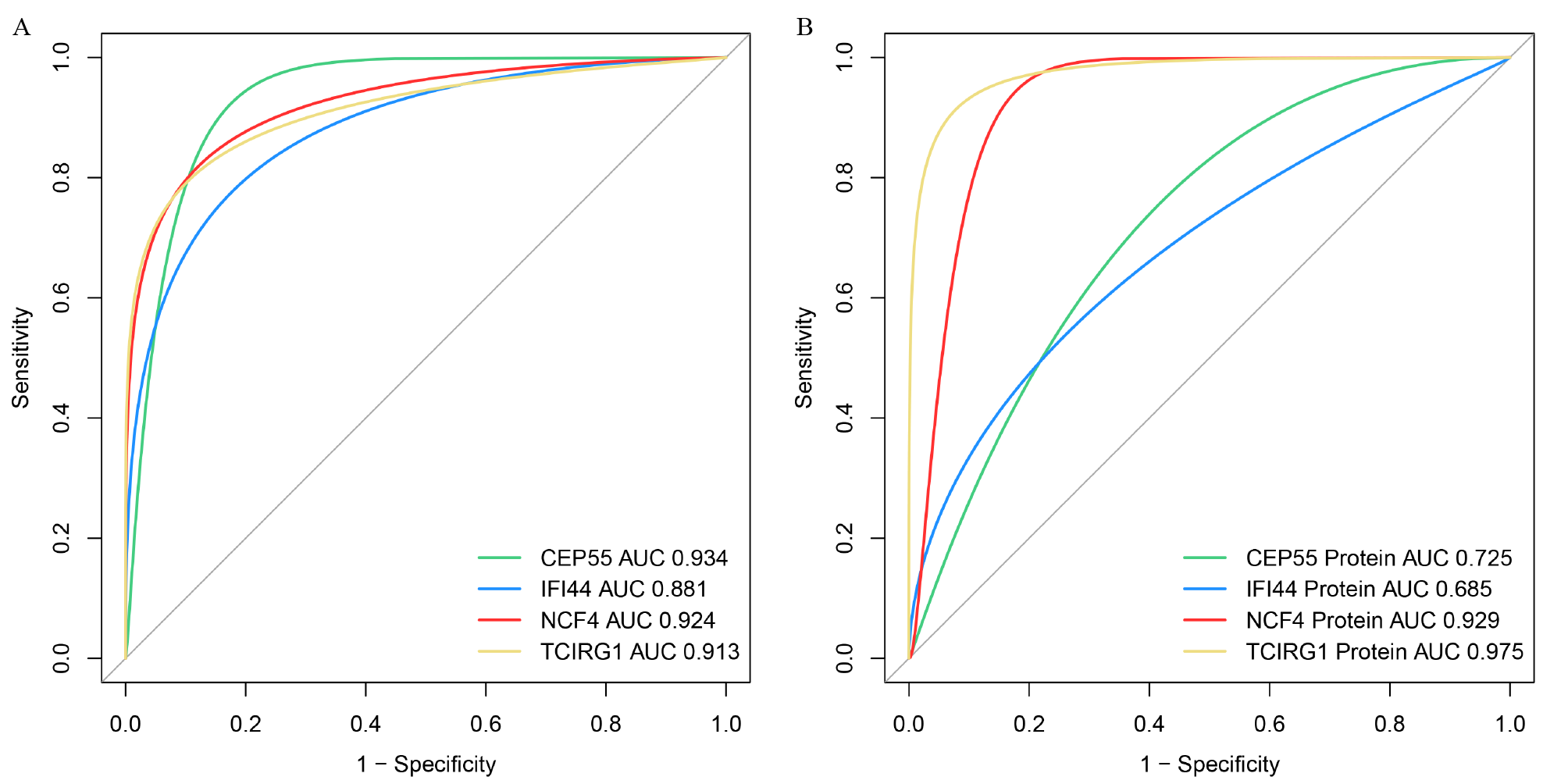

Supplement: FIGURE S6 — ROC curve analysis of CEP55, IFI44, NCF4, and TCIRG1 (A) and corresponding proteins (B) for the diagnosis of ccRCC in TCGA database. [file Image_6.TIF]

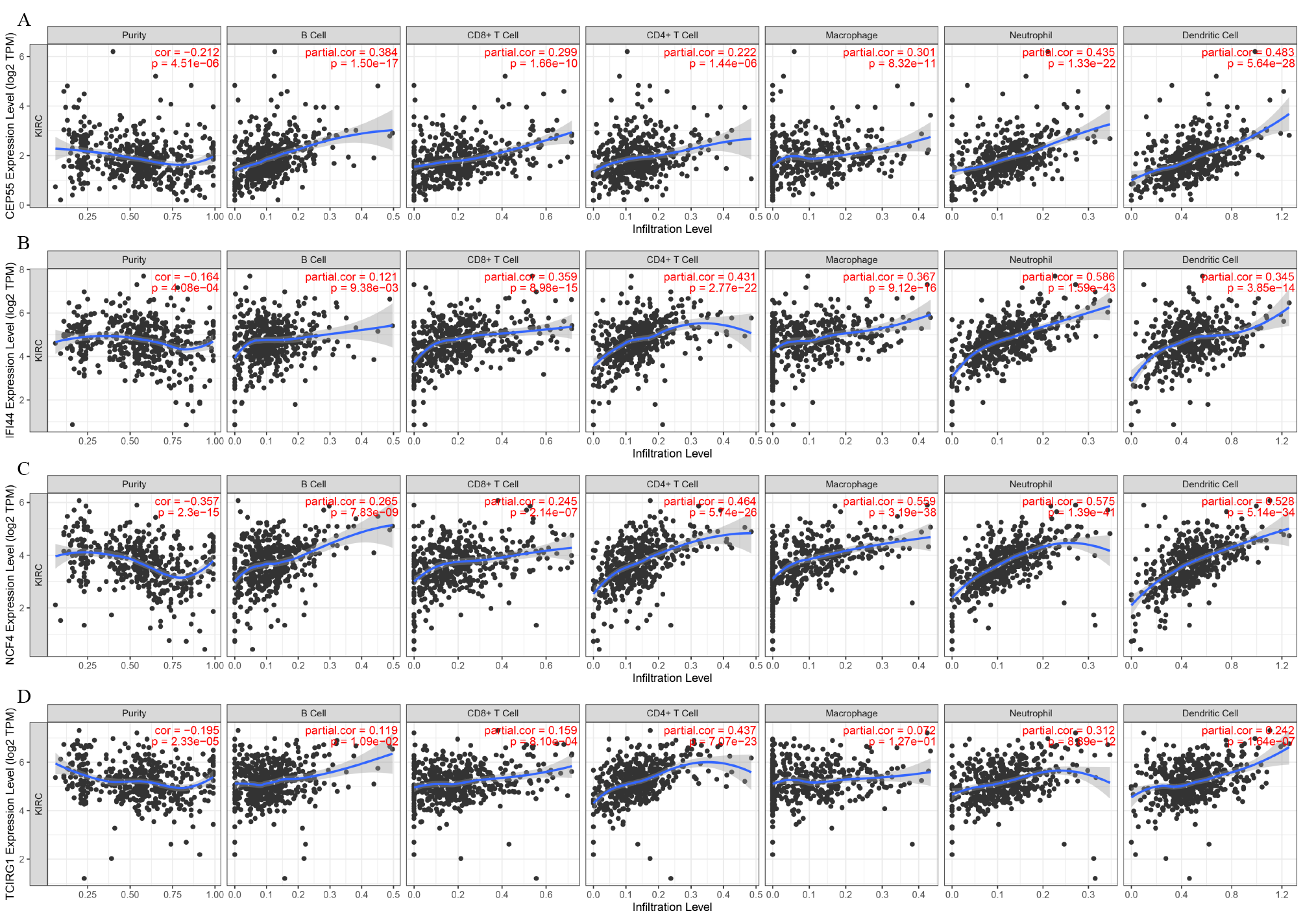

Supplement: FIGURE S7 — Association of expression of CEP55 (A), IFI44 (B), NCF4 (C), and TCIRG1 (D) with six tumor-infiltrating lymphocytes in ccRCC. P < 0.05 is regarded as statistically significant. Each dot represents a ccRCC sample in the TCGA database. [file Image_7.TIF]
