# Supplementary material for: Identification of a Novel Signature and Construction of a Nomogram Predicting Overall Survival in Clear Cell Renal Cell Carcinoma
Source: Front Genet. 2020 Sep 4;11:1017. doi: 10.3389/fgene.2020.01017 (PMC7500318; doi:10.3389/fgene.2020.01017)
Supplement: Supplementary file 8 [file Table_1.DOCX]

Table S1. Primers used in qRT-PCR.

| **ID** | **Primer Name** | **primer sequence (5'to3')** | **product length** |
| --- | --- | --- | --- |
| 1 | NCF4-F | AAAGTCTACGTGGGTGTGAAAC | 75 |
|  | NCF4-R | CAGGCTCTTCATGTAGGCGTT |  |
| 2 | TCIRG1-F | ACCATGGGCTCCATGTTCC | 131 |
|  | TCIRG1-R | GAGGCGTTGAGGTCTCTGAA |  |
| 3 | CEP55-F | ACTGTGGCTCCAAACTGCTT | 116 |
|  | CEP55-R | TCCCGCTGCTGATCATACAC |  |
| 4 | IFI44-F | AGGGAGTTGGTAAACGCTGG | 117 |
|  | IFI44-R | GGACCTCACAGGCTCACATC |  |
| 5 | GAPDH-F | AGAAGGCTGGGGCTCATTTG | 136 |
|  | GAPDH-R | GCAGGAGGCATTGCTGATGAT |  |
