# Supplementary material for: Identification of a Novel Signature and Construction of a Nomogram Predicting Overall Survival in Clear Cell Renal Cell Carcinoma
Source: Front Genet. 2020 Sep 4;11:1017. doi: 10.3389/fgene.2020.01017 (PMC7500318; doi:10.3389/fgene.2020.01017)
Supplement: Supplementary file 9 [file Table_2.DOCX]

| Table S2. Cox regression analysis of 4-mRNA signature and OS of ccRCC in internal validation cohort. | | | | |
| --- | --- | --- | --- | --- |
| Variables | Univariate analysis | | Multivariate analysis | |
|  | HR (95% CI) | *P* | HR (95% CI) | *P* |
| Age | 1.03(1.00-1.05) | 0.003 | 1.03(1.01-1.05) | 0.003 |
| T Stage |  |  |  |  |
| T1 | ref |  |  |  |
| T2 | 1.53(0.73-3.16) | 0.3 |  |  |
| T3 | 3.92(2.42-6.32) | <0.001 |  |  |
| T4 | 11.82(4.51-30.96) | <0.001 |  |  |
| N Stage |  |  |  |  |
| N0 | ref |  |  |  |
| N1 | 2.52(0.98-6.43) | 0.05 |  |  |
| NX | 0.96(0.62-1.48) | 0.8 |  |  |
| M Stage |  |  |  |  |
| M0 | ref |  |  |  |
| M1 | 4.38(2.81-6.81) | <0.001 |  |  |
| MX | 1.52(0.36-6.30) | 0.6 |  |  |
| AJCC Stage |  |  |  |  |
| Stage I | ref |  |  |  |
| Stage Ⅱ | 1.56(0.69-3.48) | 0.2 | 1.34(0.587-3.04) | 0.4 |
| Stage Ⅲ | 3.22(1.82-5.69) | <0.001 | 2.57(1.40-4.69) | 0.002 |
| Stage ⅠV | 7.07(4.14-12.07) | <0.001 | 5.79(3.16-10.61) | <0.001 |
| Grade |  |  |  |  |
| G1&G2 | ref |  |  |  |
| G3&G4 | 2.49(11.54-4.0) | <0.001 | 1.22(0.71-2.10) | 0.45 |
| Risk score |  |  |  |  |
| low | ref |  |  |  |
| high | 3.06(1.84-5.10) | <0.001 | 2.56(1.53-4.29) | <0.001 |

**Identification of a 4-mRNA Signature and Construction of a Nomogram Predicting Overall Survival in Clear Cell Renal Cell Carcinoma**

Xiangkun Wu^1, 2#^, Zhijian Zhao^1, 2#^, Aisha Khan ^3^, Chao Cai^1, 2^, Daojun Lv ^1, 2^, Di Gu^1, 2*^, Yongda Liu^1, 2*^

1 Department of Urology, Minimally Invasive Surgery Center, The First Affiliated Hospital of Guangzhou Medical University, Guangzhou, China

2 Guangdong Key Laboratory of Urology, Guangzhou Institute of Urology, Guangzhou, China

3 Department of Family Medicine, Yunshan Medical Hospital Shenzhen, China

**Current Address:** *Minimally Invasive Surgery Center, The First Affiliated Hospital of Guangzhou Medical University, Kangda Road 1#, Haizhu District, Guangzhou, Guangdong, China, 510230.

***Correspondence to:** Prof. Yongda Liu, Email: 13719007083@163.com or PhD. Di Gu, Email: di.gu@doctors.org.uk. **#**These authors contribute equally to this work as the co-first authors.

**Email for all authors:** Xiangkun Wu, [18718276810@163.com](mailto:18718276810@163.com); Zhijian Zhao, [515585921@qq.com](mailto:515585921@qq.com); Aisha Khan, [dr_aishak@hotmail.com](mailto:dr_aishak@hotmail.com); Chao Cai, [673059209@qq.com](mailto:673059209@qq.com); Daojun Lv, [15914336377@163.com](mailto:15914336377@163.com); Di Gu, di.gu@doctors.org.uk; Yongda Liu, [13719007083@163.com](mailto:13719007083@163.com).

| Table S2. Cox regression analysis of 4-mRNA signature and OS of ccRCC in the entire cohort. | | | | |
| --- | --- | --- | --- | --- |
| Variables | Univariate analysis | | Multivariate analysis | |
|  | HR (95% CI) | *P* | HR (95% CI) | *P* |
| Age | 1.03(1.01-1.04) | <0.001 | 1.03(1.01-1.04) | <0.001 |
| T Stage |  |  |  |  |
| T1 | ref |  |  |  |
| T2 | 1.51(0.90-2.51) | 0.11 |  |  |
| T3 | 3.22(2.27-4.55) | <0.001 |  |  |
| T4 | 10.71(5.40-21.22) | <0.001 |  |  |
| N Stage |  |  |  |  |
| N0 | ref |  |  |  |
| N1 | 3.6(1.91-6.79) | <0.001 |  |  |
| NX | 0.83(0.61-1.13) | 0.24 |  |  |
| M Stage |  |  |  |  |
| M0 | ref |  |  |  |
| M1 | 4.44(3.24-6.08) | <0.001 |  |  |
| MX | 0.92(0.28-2.89) | 0.88 |  |  |
| AJCC Stage |  |  |  |  |
| Stage I | ref |  |  |  |
| Stage Ⅱ | 1.21(0.64-2.23) | 0.55 | 1.05(0.56-1.97) | 0.86 |
| Stage Ⅲ | 2.54(1.68-3.83) | <0.001 | 1.80(1.17-2.77) | 0.007 |
| Stage ⅠV | 6.59(4.50-9.64) | <0.001 | 4.85(3.21-7.31) | <0.001 |
| Grade |  |  |  |  |
| G1&G2 | ref |  |  |  |
| G3&G4 | 2.72(1.92-3.83) | <0.001 | 1.66(1.15-2.40) | 0.006 |
| Risk score |  |  |  |  |
| low | ref |  |  |  |
| high | 2.88(2.05-4.04) | <0.001 | 2.49(1.76-3.50) | <0.001 |
