# Supplementary material for: Identification of a Novel Signature and Construction of a Nomogram Predicting Overall Survival in Clear Cell Renal Cell Carcinoma
Source: Front Genet. 2020 Sep 4;11:1017. doi: 10.3389/fgene.2020.01017 (PMC7500318; doi:10.3389/fgene.2020.01017)
Supplement: Supplementary file 10 [file Table_3.DOCX]

| Table 3. Cox regression analysis of 4-mRNA signature and OS of ccRCC in the entire cohort. | | | | |
| --- | --- | --- | --- | --- |
| Variables | Univariate analysis | | Multivariate analysis | |
|  | HR (95% CI) | *P* | HR (95% CI) | *P* |
| Age | 1.03(1.01-1.04) | <0.001 | 1.03(1.01-1.04) | <0.001 |
| T Stage |  |  |  |  |
| T1 | ref |  |  |  |
| T2 | 1.51(0.90-2.51) | 0.11 |  |  |
| T3 | 3.22(2.27-4.55) | <0.001 |  |  |
| T4 | 10.71(5.40-21.22) | <0.001 |  |  |
| N Stage |  |  |  |  |
| N0 | ref |  |  |  |
| N1 | 3.6(1.91-6.79) | <0.001 |  |  |
| NX | 0.83(0.61-1.13) | 0.24 |  |  |
| M Stage |  |  |  |  |
| M0 | ref |  |  |  |
| M1 | 4.44(3.24-6.08) | <0.001 |  |  |
| MX | 0.92(0.28-2.89) | 0.88 |  |  |
| AJCC Stage |  |  |  |  |
| Stage I | ref |  |  |  |
| Stage Ⅱ | 1.21(0.64-2.23) | 0.55 | 1.05(0.56-1.97) | 0.86 |
| Stage Ⅲ | 2.54(1.68-3.83) | <0.001 | 1.80(1.17-2.77) | 0.007 |
| Stage ⅠV | 6.59(4.50-9.64) | <0.001 | 4.85(3.21-7.31) | <0.001 |
| Grade |  |  |  |  |
| G1&G2 | ref |  |  |  |
| G3&G4 | 2.72(1.92-3.83) | <0.001 | 1.66(1.15-2.40) | 0.006 |
| Risk score |  |  |  |  |
| low | ref |  |  |  |
| high | 2.88(2.05-4.04) | <0.001 | 2.49(1.76-3.50) | <0.001 |
